# Evolution of the Prevalence of Antibiotic Resistance to *Staphylococcus* spp. Isolated from Horses in Florida over a 10-Year Period

**DOI:** 10.3390/vetsci10020071

**Published:** 2023-01-18

**Authors:** Kalie Marshall, Rosanna Marsella

**Affiliations:** Department of Small Animal Clinical Sciences, College of Veterinary Medicine, University of Florida, 2015 SW 16th Avenue, Gainesville, FL 32610, USA

**Keywords:** antibiotic resistance, equine, pyoderma, *Staphylococcus*

## Abstract

**Simple Summary:**

The purpose of this study was to determine how common antibiotic-resistant infections are in horses, particularly *Staphylococcus* species. These are bacteria that are normally found on the skin of horses. Overgrowth of these bacteria can lead to infection. In recent years, the emergence of resistance to antibiotics to treat these infections has been shown with these bacteria in humans and dogs. Determining how widespread *Staphylococcal* resistant bacteria are in horses helps to educate the veterinary profession on potential changes to horse resistance. This can help guide appropriate antibiotic usage as well as prove the need for innovative treatment options for both veterinary and human medicine. This study found increasing resistance in a class of antibiotics in the population observed at our institution. In addition, the species of *Staphylococcal* bacteria affects the resistance. Larger studies with more horses are needed to evaluate the clinical usefulness of these results.

**Abstract:**

Previous studies documented antibiotic resistance in horses but did not focus on skin specifically. We investigated antibiotic resistance and correlations between resistance patterns in skin infections. Records from 2009 to 2019 were searched for *Staphylococcal* infection and susceptibility results. Seventy-seven cases were included. Organisms identified were *S. aureus* (48/77), *S. pseudintermedius* (7/77), non-hemolytic *Staphylococcus* (8/77), beta-hemolytic *Staphylococcus* (6/77), and other species (8/77). Samples included pyoderma (36/77), wounds (10/77), abscesses (15/77), incision sites (5/77), nose (8/77), and foot (3/77). A trend analysis using non-parametric Spearman’s test showed significant upward trend of resistance *(p* < 0.05) for 3/15 antibiotics (ampicillin, cefazolin, penicillin). Susceptibility was significantly different by *Staphylococcal* species for 8/15 antibiotics. Gentamicin showed significant susceptibility differences based on source (all abscesses were susceptible to gentamicin). Steel-Dwass test showed statistically significant (*p* = 0.003) difference between incision sites and abscesses. A non-parametric Kendall’s T-test found significantly negative correlation between cefazolin and amikacin sensitivity (*p* = 0.0108) and multiple positive correlations of resistance (*p* < 0.05). This study confirms increasing resistance in dermatologic samples. It is unlikely that the sample source affects resistance, but *Staphylococcus* species may affect it. Study limitations include lack of information about previous antibiotic use and small sample size.

## 1. Introduction

Cutaneous bacterial infections represent a frequent, widespread challenge in equine medicine with allergic horses being at increased risk for recurrent *Staphylococcal* infections. Empiric and prophylactic use of antibiotics is widely practiced to treat these infections [1], and their overuse is threatening to diminish their value as therapeutic agents. With difficulty determining and/or controlling the underlying causes of the infections, antibiotics are frequently the mainstay of treatment. 

Evidence exists, however, that focal lesions or mild infections are likely to resolve with topical therapy alone [2,3]. Thus, the continued practice of treating mildly affected horses with systemic antibiotics leads to their overuse and the inevitable consequence of increased resistance patterns that are being seen today. Antibiotic use creates selective pressure on bacterial populations and leads to the emergence of new strains with resistance mechanisms against the action of the antibiotic used [4]. Evidence of antibiotic resistance in horses has been previously described [5,6]. The rise of increased antibiotic resistance in veterinary medicine poses a potential threat to human medicine as well as limited treatment options for practitioners, increases in morbidity, morality and cost of treatment [7,8,9]. One particular pathogenic genus that is capable of developing resistance readily is *Staphylococcus* [10,11,12].

Staphylococci are common opportunistic pathogens isolated most frequently from equine skin infections. A wide variety of *Staphylococcal* species are part of the equine commensal microbiota and can be found on healthy horse skin [13], but once integumentary barriers are breached, they cause infection. The greatest cause for concern with staphylococci is their tendency to become resistant to antibiotics. This resistance has caused much concern for a variety of reasons, such as being more difficult to treat and the prospect of zoonotic transmission in which humans can acquire resistance from horses [13]. 

Methicillin-resistance occurs when staphylococci acquire a genetic mutation through horizontal transfer. These bacteria carry a copy of the *mecA* gene that encodes penicillin-binding proteins with low affinity for beta-lactam antibiotics [14]. Beta-lactam antibiotics normally inhibit bacterial cell wall biosynthesis through irreversible binding to penicillin-binding proteins, but with this mutation the bacteria gain resistance [15]. Treating methicillin resistance can be increasingly difficult because it is often paralleled by resistance to other antibiotics [16]. In addition, horses are already limited in the number of antibiotics they can tolerate due to their delicate hindgut so increasing resistance limits treatment options further for practitioners. 

Coagulase-positive species (*S. aureus*, *S. pseudintermedius*, *S. delphini*, and *S. hyicus*) are the most important pathogens clinically [17,18]. Coagulase-negative staphylococci are normally less virulent and express fewer virulence factors [17,18], however, coagulase-negative species may also result in infection in immunocompromised animals [13]. *Staphylococcus aureus* has been reported as one of the most common causes of *Staphylococcal* disease in horses [19,20]. With the rise of methicillin-resistance in *Staphylococcal* species, there is a greater concern for horses being a reservoir for methicillin-resistant *S. aureus* (MRSA) [21]. MRSA and methicillin-resistant coagulase-negative staphylococci (MRCoNS) are a major problem for human and animal populations [21,22] thus making monitoring of resistance extremely important.

The threat of antibiotic resistant (AMR) bacterial infections has been internationally recognized for decades [23]. However, to the authors’ knowledge, very few studies have followed developments of patterns in the equine species. The objective of this study was to determine if there was an increasing trend in antibiotic resistance in the equine species over a 10-year period at a veterinary teaching hospital. Secondary objectives were to report the prevalence of resistance seen for individual antibiotics during the 10-year time frame as well as to report correlations between resistance patterns of specific antibiotics.

## 2. Materials and Methods

The clinical records of all horses presented to the authors’ academic institution from 1 January 2009 to 1 October 2019 were retrospectively and electronically searched using the key words “aerobic culture” and “dermatology culture”. Inclusion criteria were the presence of *Staphylococcal* infection, susceptibility panel results, and samples taken from pyoderma lesions, surgical incision sites, superficial wounds, abscesses, the nose, or foot.

For each case, the following information was retrieved: location of the sample site, organism cultured, date of culture, and results of the susceptibility panel. 

Samples included in the study were plated on blood agar, cultured at 37 °C overnight and observed for growth the following day. Bacterial pathogens were identified based on biochemical reactions using the Sensititre ARIS 2X (TREK Diagnostic Systems Inc.; Cleveland, OH, USA) automated system. All isolates were tested according to the manufacturers’ recommendations using the Sensititre Gram-positive and Gram-negative identification panels incubated and auto-read by the ARIS 2X System. 

The minimum inhibitory concentrations (MICs) of erythromycin, gentamicin, imipenem, oxacillin, rifampin, amikacin, ampicillin, azithromycin, cefazolin, chloramphenicol, clarithromycin, doxycycline, penicillin, tetracycline, and trimethoprim/sulfonamide were determined using the broth microdilution Sensititre automated system according to the manufacturers’ instructions. The 2004 Clinical and Laboratory Standards Institute (CLSI) breakpoints were used for the MICs for samples collected from 2009 to 2013 [24]. The 2013 CLSI breakpoints were used for samples in 2014 and 2015 and the 2015 CLSI breakpoints were used for samples collected from 2016 to 2019 [25,26].

Association between antibiotic susceptibility and *Staphylococcal* organism was measured using the Kruskal–Wallis rank sum test for each antibiotic separately. The cut-off *p*-values were set at 0.05 to indicate statistical significance. For the antibiotics showing a significant difference, the Steel-Dwass method was used to compare the antibiotic susceptibility between each *Staphylococcal* organism. The Kruskal–Wallis rank sum test was also used to measure the association between sample collection sites and antibiotic susceptibility. The cut-off *p*-values were set at 0.05 to indicate statistical significance. For the antibiotics showing a significant difference, the Steel-Dwass method was used to compare the antibiotic susceptibility between each sample collection site. A trend analysis was also completed using a nonparametric Spearman’s test comparing date of sample selection to susceptibility. Finally, a nonparametric Kendall’s τ test was used to analyze the correlations between each antibiotic and the Kendall’s τ value was reported. The cut-off p-value of 0.05 was reported to indicate statistical significance. All statistics were performed using the JMP software program (JMP®, Version 15; SAS Institute Inc., Cary, NC, USA).

## 3. Results

Of the 2300 aerobic and dermatology cultures collected on horses in the 10-year time frame, 77 *Staphylococcus* samples met the inclusion criteria. *Staphylococcus aureus* was the most prevalent sample isolated (48/77; 62.3%). The other isolates were identified as follows: *S. pseudintermedius* (7/77; 9.1%), *S. hyicus* (2/77; 2.6%), *S. xylosus* (2/77; 2.6%), *S. epidermidis* (2/77; 2.6%), and *S. schleiferi* (1/77; 1.3%). The remainder of the samples were identified as *Staphylococcus*, but the species was not reported. They were reported as non-hemolytic *Staphylococcus* (8/77; 10.4%), beta-hemolytic *Staphylococcus* (6/77; 7.8%), and *Staphylococcus* spp. (1/77; 1.3%). These organisms were grouped into five categories for data analysis *S. aureus*, *S. pseudintermedius*, non-hemolytic *Staphylococcus*, beta-hemolytic *Staphylococcus*, and the miscellaneous group (MG), which consisted of the rest of the individual species isolated. 

All cultured organisms were tested against erythromycin, gentamicin, imipenem, oxacillin and rifampin (77/77). Other antibiotics tested were amikacin (66/77), ampicillin (68/77), azithromycin (76/77), cefazolin (75/77), chloramphenicol (76/77), clarithromycin (60/77), doxycycline (51/77), penicillin (67/77), tetracycline (75/77), and trimethoprim/sulfonamide (TMS) (76/77).

Of the 77 samples, the majority came from pyoderma lesions (36/77; 46.8%). The other locations of sample selection included superficial wounds (10/77; 13%), abscesses (15/77; 19.5%), surgical incision sites (5/77; 6.5%), the nose (8/77; 10.4%), and the foot (3/77; 3.9%). Using the Kruskal–Wallis rank sum test, sites of sample collection were compared to each antibiotic susceptibility. Gentamicin was the only antibiotic that showed a significant difference in susceptibility based on the sample collection site. Therefore, data points for gentamicin were analyzed using the Steel-Dwass method to determine which sample type contained *Staphylococcus* that were more resistant or susceptible. It was found that all 15 *Staphylococcus* spp. isolated from abscesses were susceptible to gentamicin. However, 70% of *Staphylococcus* spp. isolated from surgical incision sites, 33.3% isolated from the foot, 30.5% isolated from pyoderma lesions, 30% isolated from superficial wounds, and 12.5% isolated from the nose were resistant to gentamicin. Statistical significance was achieved (*p* = 0.003) in the Steel-Dwass test when comparing surgical incision site to abscesses (Table 1). The comparison of pyoderma lesions to abscesses was not statistically significant (*p* = 0.1225).

To investigate an association between year of sample isolation and resistance patterns, a trend analysis was completed using a nonparametric Spearman’s test. The results indicated that 3/15 antibiotics tested over the 10-year period showed a significant (*p* = 0.05) upward trend of resistance (Table 2). 

Using the Kruskal–Wallis rank sum test, *Staphylococcal* isolate groups were compared to each antibiotic susceptibility. Results of 8/15 antibiotics tested showed a significant difference in susceptibility based on the isolate group. These eight antibiotics were then analyzed with the Steel-Dwass test to determine which organisms demonstrated greater resistance and which organisms followed similar resistance patterns for each antibiotic. Ampicillin, azithromycin, cefazolin, clarithromycin, erythromycin, imipenem, oxacillin and penicillin all had at least one *Staphylococcal* isolate more likely to be resistant than the others. The MG consisting of *S. hyicus*, *S. xylosus*, *S. epidermidis*, *S. schleiferi*, and isolates reported as *Staphylococcus* spp. showed statistical significance that they were more likely to be resistant to ampicillin, azithromycin, cefazolin, erythromycin, imipenem, oxacillin, and penicillin than *S. aureus*. The *S. pseudintermedius* group showed equal likelihood to be resistant to ampicillin and penicillin as *S. aureus*. The non-hemolytic *Staphylococcus* group was more likely to be resistant to azithromycin, clarithromycin, erythromycin, and oxacillin than the *S. aureus* group. The MG was more likely to be resistant to cefazolin than the beta-hemolytic *Staphylococcus* group. There were also numerous organism groups that showed very similar resistance prevalence to one another for each antibiotic. These similarities can be seen in Table 3 along with the statistics for the aforementioned relationships. The organism groups with a *p*-value close to one demonstrate a similar likelihood to be resistant to that particular antibiotic. 

Additionally, a nonparametric Kendall’s τ test was used to analyze the correlations between each antibiotic. Many statistically significant positive correlations were found between antibiotics. Only one significant negative correlation was found between cefazolin and amikacin. Strong positive correlations of susceptibility were found between multiple antibiotics and can be seen in the supporting information (Supplemental Table S1). Finally, the overall prevalence of resistance or intermediate susceptibility for seven antibiotics was determined for each of the organism groups in the 10-year time period (Table 4 and Table 5).

## 4. Discussion

The results of this study suggest that resistance for certain antibiotics is present and patterns are being seen across antibiotic classes. Ampicillin, cefazolin, and penicillin showed a statistically significant upward trend of resistance over the 10-year period examined. Since these antibiotics are not commonly used by dermatologists, this could be due to their increased use for infections not related to the skin. Penicillin is frequently used for surgical prophylaxis in horses undergoing colic surgery [1] and is used as a first line choice for treatment of streptococcal infections, the cause of equine strangles and upper and lower respiratory infections in horses [27]. Ampicillin is used in horses for streptococcal lower airway infections in addition to being a treatment for neonatal sepsis, while cefazolin is a treatment choice for culture-confirmed susceptible *Staphylococcus* spp. [27]. All of these uses could be leading to the increased resistance seen in this study. It is important to note that other horses in the same pasture or boarding facility may have been treated previously and created a selective pressure on the bacteria in the area [4]. 

Blood supply of skin is comparatively poor to other organs and the upper end of dose ranges are used to treat skin infections. To be effective, antibiotics must reach sufficient concentrations at the site of infection. Tissue distribution is an important factor for antibiotic efficacy and due to the challenges of achieving these concentrations in the epidermis, systemic treatments for superficial pyoderma involve larger doses and longer durations compared to treatments for other infections. These factors may play a role in why dermatology cases tend to develop more resistance than other patients. Some antibiotics, such as rifampin, macrolides, and fluoroquinolones, are lipophilic, thus accumulate in larger amounts in the skin [28]. This paper focused specifically on how antibiotic sensitivity in vitro has changed over time and when translating these results to clinical practice it is important to put them into context of the characteristics of the specific antibiotic.

Trimethoprim/sulfonamide also showed an increasing trend of resistance that did not reach statistical significance, but may be of clinical relevance as this is often a first choice for systemic treatment of horses with skin infections due to the cost and spectrum of activity of this class of antibiotic [29,30]. In a Canadian study, trimethoprim/sulfonamide’s increased resistance had been shown previously in coagulase-positive staphylococci [6]. However, another study in France showed that TMS resistance was stationary over a four-year time period [31]. Therefore, further investigation is warranted. It is possible that TMS would have reached statistical significance in this study if a longer period of time was analyzed or if the study started before 2009 due to the fact that the population of bacteria could have already matured or developed resistance prior to the start of the study. Alternatively, the bacterial population in different geographical locations potentially have different resistance patterns in part due to the distinct ways medicine is practiced or the different antibiotics used in various locations leading to unique pressure on the bacteria.

The low numbers of oxacillin resistance of all *Staphylococcal* species in this study is in contrast to what is seen in small animal medicine in which an increase has been seen in oxacillin resistance in dogs in recent years [32,33,34]. However, the low prevalence of oxacillin resistance seen in this study is similar to that seen in livestock [35]. It is hypothesized that there is less methicillin/oxacillin resistance in livestock and horses when compared to small animals due to the increased regulations on antibiotic use and decreased available antibiotics for use in the large animal species. A study in the Netherlands showed decreased antibiotic use was associated with declining methicillin resistance [36].

It is possible that some of the resistance documented in penicillin, ampicillin, and cefazolin is due to increased numbers of staphylococci producing beta lactamase and not due to an acquired *mecA* gene mutation as is required for methicillin resistance. The semi-synthetic penicillins can circumvent beta lactamase activity [37]. This could explain why some of the beta-lactam antibiotics have increased resistance. In addition, the use of fluoroquinolones is likely higher in dogs than horses due to the cost. A previous study in Germany reported the use of fluoroquinolones to have been less than 1% of antibiotics administered or dispensed to horses [38]. Fluoroquinolones have been shown to increase the risk of antibiotic resistance in humans and dogs [39,40,41].

Moreover, gentamicin was the only antibiotic that showed a statistical difference in susceptibility based on the sample collection type. The difference was found between the surgical incision sites and the abscesses while all other sample types showed no statistical difference. It was hypothesized that there would be no difference in susceptibility based on sample type and this difference could potentially be due to small sample sizes. There were only five samples in the surgical incision group and 15 in the abscess group. However, all of the abscesses were susceptible to gentamicin while only one of the surgical incision sites was susceptible. Further investigation with larger sample sizes would be warranted to confirm sample type affects susceptibility. 

There were many significant positive correlations found between the antibiotics that were analyzed and one significant negative correlation (Supplemental Table S1). Many of the positive correlations were between antibiotics in the same drug class which was expected due to their similar mechanisms of action. In addition, many other positive correlations were found between antibiotics in the same tier, such as rifampin and chloramphenicol. This was also to be anticipated due to the similar amount of use these antibiotics get causing similarities in selective pressure. The single negative correlation was between cefazolin and amikacin and is likely not of clinical relevance. 

Lastly, the *Staphylococcal* species often did affect the antibiotic susceptibility as expected. Historically, coagulase negative staphylococci have been more resistant to antibiotics than coagulase positive staphylococci [42]. This aligns with the current study where the “other” group, which consisted of *S. hyicus*, *S. xylosus*, *S. epidermidis*, and *S. schleiferi*, showed a statistical significance of being more resistant to beta-lactam antibiotics and macrolides when compared to *S. aureus*. In addition, *S. aureus* and *S. pseudintermedius*, both coagulase positive staphylococci, showed equal likelihood to be resistant to ampicillin and penicillin. From a clinical point of view, this data is important as most pyodermas that dermatologists treat are coagulase positive staphylococci. 

As previously mentioned, one of the main limitations of this study is a small sample size. The numbers in this study are likely an underestimation of the population of horses treated at the author’s academic institution. This is due to the retrospective nature of the study and the difficulty associated with searching medical record systems. Moreover, the previous antibiotic history was unknown or not reported in a majority of the cases. Of the 77 cases included, 49 had an unknown antibiotic history and 24 had at least one antibiotic used prior to being cultured. We suspect that there is a selection bias in this study due to the fact that culture and sensitivity panels are often submitted after a patient is not responding to an empiric treatment or after previously being treated with antibiotics. Therefore, higher resistance was expected. In addition, this can be assumed due to the tertiary referral nature of the institution being studied. Many of the patients at the institution have had chronic infections that could not be cleared by their primary veterinarian or the first line antibiotic was not working. To end, this study is important to help show the progress of bacterial resistance and guide field veterinarians on their empiric choices. Continual monitoring of resistance patterns is essential for better antibiotic use and maximizes the chance of successful therapy.

## 5. Conclusions

This study suggests that resistance of beta-lactam antibiotics for *Staphylococcus* is on the rise at the author’s tertiary referral institution, the specific *Staphylococcal* spp. isolated affects resistance and antibiotics in similar classes are likely to follow similar resistance patterns. Further larger-sample studies are needed to assess the clinical usefulness of the aforementioned results.

## Figures and Tables

**Table 1:** Susceptible *Staphylococcal* species for each sample collection site.

	Pyoderma		Superficial Wounds		Abscesses		Surgical Incision Sites		Nose		Foot	
	N	%	N	%	N	%	N	%	N	%	N	%
**Erythromycin**	28/36	77.8	8/10	80	13/15	86.7	3/5	60	6/8	75	1/3	33.3
**Gentamicin**	24/36	66.7	7/10	70	15/15	100	1/5	20	7/8	87.5	2/3	66.7
**Imipenem**	30/36	83.3	8/10	80	12/15	80	3/5	60	5/8	62.5	1/3	33.3
**Oxacillin**	25/30	83.3	7/9	77.8	11/14	78.6	3/5	60	5/8	62.5	1/3	33.3
**Rifampin**	36/36	100	9/10	90	14/15	93.3	5/5	100	8/8	100	3/3	100
**Amikacin**	23/28	82.1	8/10	80	13/14	92.9	1/2	50	6/6	100	2/2	100
**Ampicillin**	20/32	62.5	5/10	50	12/15	80	2/4	50	3/6	50	1/1	100
**Azithromycin**	29/36	80.6	9/10	90	12/14	85.7	3/5	60	6/8	75	1/3	33.3
**Cefazolin**	15/35	42.9	3/10	30	8/15	53.3	2/5	40	4/7	57.1	1/3	33.3
**Chloramphenicol**	34/36	94.4	8/10	80	15/15	100	5/5	100	7/7	100	2/3	66.7
**Clarithromycin**	22/28	78.6	8/9	88.9	11/13	84.6	1/3	33.3	4/6	66.7	1/1	100
**Doxycycline**	21/22	95.5	5/6	83.3	13/14	92.9	2/2	100	5/6	83.3	1/1	100
**Penicillin**	18/32	56.3	5/10	50	11/14	78.6	2/4	50	3/6	50	1/1	100
**Tetracycline**	29/36	80.6	6/9	66.7	13/14	92.9	3/5	60	7/8	87.5	3/3	100
**TMS**	26/36	72.2	6/9	66.7	13/15	86.7	3/5	60	5/8	62.5	2/3	66.7

Number and percentage of susceptible *Staphylococcal* species for each sample collection site examined during the time period 1 January 2009–1 October 2019 at the author’s institution. Gentamicin was the only antibiotic showing statistical significance based on sample collection site. This occurred between abscesses and surgical incision sites and the percent susceptible for each site are highlighted in yellow. TMS, trimethoprim/sulfonamide.

**Table 2:** Antibiotic resistance over a ten-year time period.

Variable	by Variable	Spearman ρ	Prob > |ρ|	Difference Plot
Date	Amikacin	–0.1582	0.2234	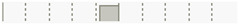
Date	Ampicillin	0.2649	0.0290	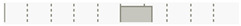
Date	Azithromycin	–0.0212	0.8559	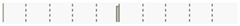
Date	Cefazolin	0.3012	0.0086	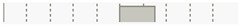
Date	Chloramphenicol	–0.1176	0.3116	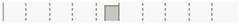
Date	Clarithromycin	0.1034	0.4316	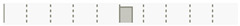
Date	Doxycycline	–0.0842	0.5567	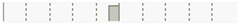
Date	Erythromycin	0.0135	0.9070	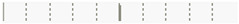
Date	Gentamycin	0.0635	0.5831	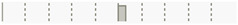
Date	Imipenem	–0.1567	0.1735	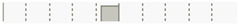
Date	Oxacillin	–0.1013	0.4074	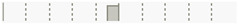
Date	Penicillin	0.2541	0.0380	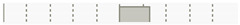
Date	Rifampin	–0.0404	0.7271	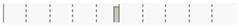
Date	Tetracycline	0.0379	0.7466	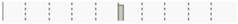
Date	TMS	0.1697	0.1428	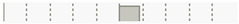

Test results for association between date of sample collection and resistance for each antibiotic. Antibiotics highlighted in yellow show an upward trend over the last 10 years. The *p* values highlighted in red illustrate the significant trends. The difference plots show a measure of strength and direction of association using the grey bars in the far-right column.

**Table 3:** Results for association between *Staphylococcal* isolate groups and antibiotic resistance.

**Ampicillin**								
**IG**	**-IG**	**Score Mean Difference**	**Std Err Dif**	**Z**	***p*** **-Value**	**Lower** **CL**	**Upper** **CL**	**Difference Plot**
3	1	13.9167	4.871794	2.85658	0.0348 *	0.000	1.000	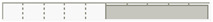
4	1	0.0000	4.565305	0.00000	1.000	–1.0000	1.000	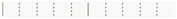
**Azithromycin**								
**IG**	**-IG**	**Score Mean Difference**	**Std Err Dif**	**Z**	***p*** **-Value**	**Lower ** **CL**	**Upper ** **CL**	**Difference Plot**
2	1	13.0952	4.160561	3.14747	0.0142 *	0.000	1.000	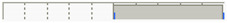
3	1	11.3021	3.968627	2.84786	0.0356 *	0.000	1.000	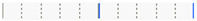
5	1	1.6875	3.712311	0.45457	0.9912	0.000	0.500	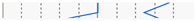
3	2	–0.4018	2.004459	–0.20045	0.9996	–1.000	1.000	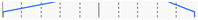
5	4	–0.6190	1.585838	–0.39036	0.9951	–1.000	1.000	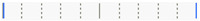
4	3	–1.4732	1.968340	–0.74846	0.9450	–1.000	1.000	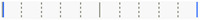
**Cefazolin**								
**IG**	**-IG**	**Score Mean Difference**	**Std Err Dif**	**Z**	***p*** **-Value**	**Lower** **CL**	**Upper ** **CL**	**Difference Plot**
3	1	18.0652	5.672817	3.18451	0.0126 *	0.000	1.000	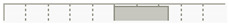
5	3	–5.6875	2.075498	–2.74031	0.0483 *	–1.000	0.000	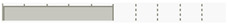
**Clarithromycin**								
**IG**	**-IG**	**Score Mean Difference**	**Std Err Dif**	**Z**	***p*** **-Value**	**Lower ** **CL**	**Upper ** **CL**	**Difference Plot**
2	1	11.8611	3.500000	3.38889	0.0063 *	0.000	1.000	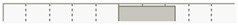
5	4	0.0000	1.610235	0.00000	1.0000	.	.	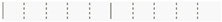
5	3	–0.7857	1.739253	–0.45175	0.9914	.	.	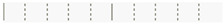
4	3	–0.8571	1.860521	–0.46070	0.9907	–1.000	1.000	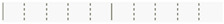
3	2	–0.9286	1.947220	–0.47687	0.9895	–1.000	1.000	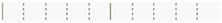
**Erythromycin**								
**IG**	**-IG**	**Score Mean Difference**	**Std Err Dif**	**Z**	***p*** **-Value**	**Lower ** **CL**	**Upper ** **CL**	**Difference Plot**
2	1	14.5104	4.132282	3.51148	0.0041 *	0.000	1.000	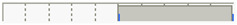
3	1	14.1458	4.139495	3.41729	0.0057 *	0.000	1.000	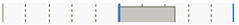
5	1	1.5938	3.708735	0.42973	0.9929	0.000	0.000	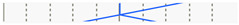
3	2	–0.5000	2.091650	–0.23905	0.9993	–1.000	1.000	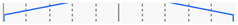
5	4	–0.6190	1.585838	–0.39036	0.9951	–1.000	1.000	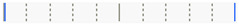
**Imipenem**								
**IG**	**-IG**	**Score Mean Difference**	**Std Err Dif**	**Z**	***p*** **-Value**	**Lower ** **CL**	**Upper ** **CL**	**Difference Plot**
3	1	17.4271	4.427189	3.93638	0.0008 *	0.000	1.0000	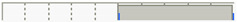
**Oxacillin**								
**IG**	**-IG**	**Score Mean Difference**	**Std Err Dif**	**Z**	***p*** **-Value**	**Lower ** **CL**	**Upper ** **CL**	**Difference Plot**
3	1	20.6250	4.491474	4.59203	<0.0001 *	0	1.000	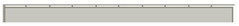
2	1	18.3292	4.720480	3.88290	0.0010 *	0	1.000	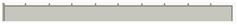
3	2	0.1714	1.367527	0.12536	0.9999	.	.	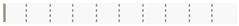
4	2	–1.1250	1.509346	–0.74536	0.9458	.	.	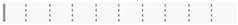
5	1	–2.6500	3.674889	–0.72111	0.9517	0	0.000	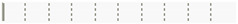
**Penicillin**								
**IG**	**-IG**	**Score Mean Difference**	**Std Err Dif**	**Z**	***p*** **-Value**	**Lower ** **CL**	**Upper ** **CL**	**Difference Plot**
3	1	17.4167	4.923900	3.53717	0.0037 *	0.000	1.000	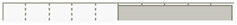
4	1	0.0000	4.565305	0.00000	1.0000	–1.000	1.000	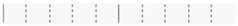
5	4	–0.3429	1.588923	–0.21578	0.9995	.	.	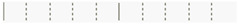
5	1	–1.9024	5.026786	–0.37845	0.9957	–1.000	1.000	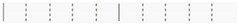

Test results for association between *Staphylococcal* isolate groups and antibiotic resistance. Group 1: *S. aureus*, Group 2: non-hemolytic *Staphylococcus*, Group 3: Miscellaneous group (MG), Group 4: *S. pseudintermedius*, Group 5: beta-hemolytic *Staphylococcus.* IG = isolate group. The *p* values marked with * illustrate the significant trends. Those that are highlighted in red have a *p* value < 0.05 and those that are highlighted in orange have a *p* value < 0.01. The difference plots show a measure of strength and direction of association using the grey bars in the far-right column.

**Table 4:** Resistant *Staphylococcal* species over the ten-year period studied.

Antibiotic	*Staphylococcus aureus*		Non-Hemolytic *Staphylococcus*		MG		*Staphylococcus pseudintermedius*		Beta-Hemolytic *Staphylococcus*	
	N	%	N	%	N	%	N	%	N	%
Oxacillin	5/48	10.4	4/5	80	6/7	85.7	2/4	50	0/5	0
Penicillin	12/42	28.6	5/6	83.3	7/7	100	2/7	28.6	1/5	20
Ampicillin	12/42	28.6	4/7	57.1	6/7	85.7	2/7	28.6	1/5	20
TMS	12/47	25.5	3/8	37.5	3/8	37.5	2/7	28.6	1/6	16.7
Cefazolin	5/47	10.6	4/8	50	6/8	75	1/6	16.7	0/6	0
Tetracycline	10/48	20.8	1/6	16.7	1/8	12.5	1/7	14.3	1/6	16.7
Rifampin	1/48	2.1	0/8	0	0/8	0	0/7	0	1/6	16.7

Number and percentage of resistant *Staphylococcal* species based on antibacterial agents tested during the time period 1 January 2009–1 October 2019 at the author’s institution. MG = miscellaneous group.

**Table 5:** *Staphylococcal* species with intermediate results over the ten-year period studied.

Antibiotic	*Staphylococcus aureus*		Non-Hemolytic *Staphylococcus*		MG		*Staphylococcus pseudintermedius*		Beta-Hemolytic *Staphylococcus*	
	N	%	N	%	N	%	N	%	N	%
Cefazolin	18/47	38.3	3/8	37.5	1/8	12.5	3/6	50	1/6	16.7

Number and percentage of *Staphylococcal* strains with intermediate results for cefazolin during the time period 1 January 2009–1 October 2019 at the author’s institution. MG = miscellaneous group.

## Data Availability

Not applicable.

## Supplementary Materials

The following supporting information can be downloaded at: https://www.mdpi.com/article/10.3390/vetsci10020071/s1, Table S1: Test results reporting the correlations between each antibiotic. Numbers listed in orange (*p* < 0.01) or red (*p* < 0.05) reached statistical significance. Many statistically significant positive correlations were found between antibiotics. Only one significant negative correlation was found between cefazolin and amikacin.
