# Supplementary material for: Evolution of the Prevalence of Antibiotic Resistance to Staphylococcus spp. Isolated from Horses in Florida over a 10-Year Period
Source: Vet Sci. 2023 Jan 18;10(2):71. doi: 10.3390/vetsci10020071 (PMC9959586; doi:10.3390/vetsci10020071)

## Supplemental

## Table S1.

| Variable | by Variable | Kendall τ | Prob>\|τ\| |
|---|---|---|---|
| Ampicillin | Amikacin | 0.0625 | 0.6347 |
| Azithromycin | Amikacin | -0.0682 | 0.5948 |
| Azithromycin | Ampicillin | 0.4384 | 0.0003* |
| Cefazolin | Amikacin | -0.3124 | 0.0108* |
| Cefazolin | Ampicillin | 0.5790 | <.0001* |
| Cefazolin | Azithromycin | 0.3943 | 0.0003* |
| Chloramphenicol | Amikacin | 0.0968 | 0.4510 |
| Chloramphenicol | Ampicillin | 0.2105 | 0.0855 |
| Chloramphenicol | Azithromycin | 0.2700 | 0.0185* |
| Chloramphenicol | Cefazolin | 0.0637 | 0.5602 |
| Clarithromycin | Amikacin | -0.0716 | 0.5796 |
| Clarithromycin | Ampicillin | 0.3776 | 0.0041* |
| Clarithromycin | Azithromycin | 0.9525 | <.0001* |
| Clarithromycin | Cefazolin | 0.2817 | 0.0229* |
| Clarithromycin | Chloramphenicol | 0.2703 | 0.0381* |
| Doxycycline | Amikacin | 0.0756 | 0.5933 |
| Doxycycline | Ampicillin | 0.3746 | 0.0094* |
| Doxycycline | Azithromycin | 0.5630 | <.0001* |
| Doxycycline | Cefazolin | 0.2529 | 0.0627 |
| Doxycycline | Chloramphenicol | 0.2359 | 0.0986 |
| Doxycycline | Clarithromycin | 0.5322 | 0.0002* |
| Erythromycin | Amikacin | -0.0911 | 0.4739 |
| Erythromycin | Ampicillin | 0.4344 | 0.0003* |
| Erythromycin | Azithromycin | 0.9281 | <.0001* |
| Erythromycin | Cefazolin | 0.4346 | <.0001* |
| Erythromycin | Chloramphenicol | 0.3429 | 0.0026* |
| Erythromycin | Clarithromycin | 0.9550 | <.0001* |
| Erythromycin | Doxycycline | 0.5058 | 0.0003* |
| Gentamicin | Amikacin | 0.5989 | <.0001* |
| Gentamicin | Ampicillin | 0.4175 | 0.0005* |
| Gentamicin | Azithromycin | 0.3343 | 0.0031* |
| Gentamicin | Cefazolin | 0.1038 | 0.3381 |
| Gentamicin | Chloramphenicol | 0.0954 | 0.3976 |
| Gentamicin | Clarithromycin | 0.2352 | 0.0634 |
| Gentamicin | Doxycycline | 0.1686 | 0.2290 |
| Gentamicin | Erythromycin | 0.2865 | 0.0107* |
| Imipenem | Amikacin | 0.0075 | 0.9533 |
| Imipenem | Ampicillin | 0.6376 | <.0001* |
| Imipenem | Azithromycin | 0.4619 | <.0001* |
| Imipenem | Cefazolin | 0.6767 | <.0001* |
| Imipenem | Chloramphenicol | 0.1097 | 0.3384 |
| Imipenem | Clarithromycin | 0.3193 | 0.0135* |
| Imipenem | Doxycycline | 0.3790 | 0.0074* |
| Imipenem | Erythromycin | 0.4820 | <.0001* |
| Imipenem | Gentamicin | 0.2109 | 0.0617 |
| Oxacillin | Amikacin | -0.1328 | 0.3344 |
| Oxacillin | Ampicillin | 0.6342 | <.0001* |

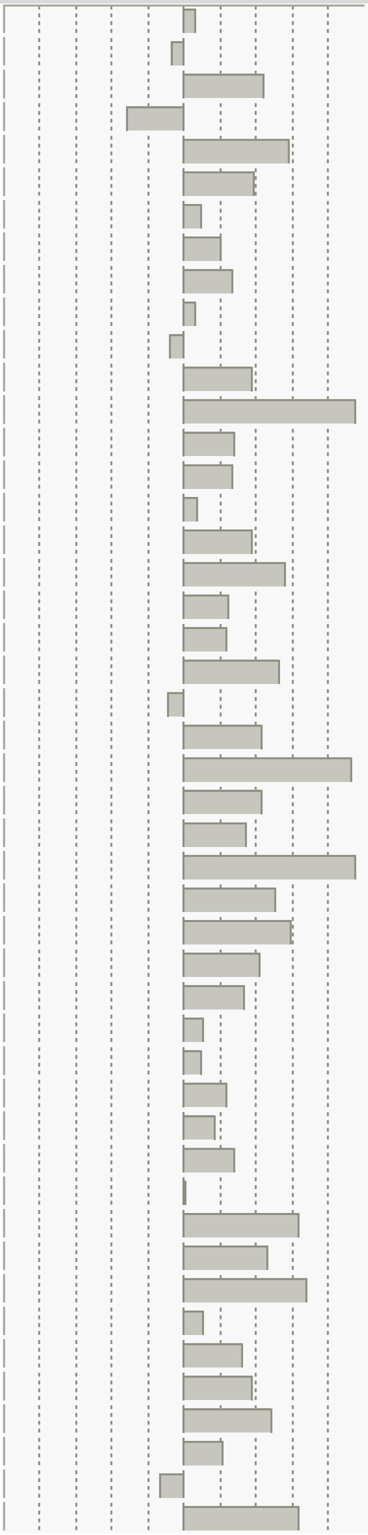

| Variable | by Variable | Kendall τ | Prob>\|τ\| |
|---|---|---|---|
| Oxacillin | Azithromycin | 0.5744 | <.0001* |
| Oxacillin | Cefazolin | 0.7607 | <.0001* |
| Oxacillin | Chloramphenicol | 0.1548 | 0.2018 |
| Oxacillin | Clarithromycin | 0.4733 | 0.0007* |
| Oxacillin | Doxycycline | 0.3965 | 0.0085* |
| Oxacillin | Erythromycin | 0.6226 | <.0001* |
| Oxacillin | Gentamicin | 0.1508 | 0.2054 |
| Oxacillin | Imipenem | 0.9624 | <.0001* |
| Penicillin | Amikacin | 0.0282 | 0.8315 |
| Penicillin | Ampicillin | 0.9391 | <.0001* |
| Penicillin | Azithromycin | 0.5639 | <.0001* |
| Penicillin | Cefazolin | 0.5510 | <.0001* |
| Penicillin | Chloramphenicol | 0.1899 | 0.1237 |
| Penicillin | Clarithromycin | 0.5050 | 0.0001* |
| Penicillin | Doxycycline | 0.3419 | 0.0191* |
| Penicillin | Erythromycin | 0.5449 | <.0001* |
| Penicillin | Gentamicin | 0.3746 | 0.0019* |
| Penicillin | Imipenem | 0.5972 | <.0001* |
| Penicillin | Oxacillin | 0.6102 | <.0001* |
| Rifampin | Amikacin | 0.2040 | 0.1116 |
| Rifampin | Ampicillin | 0.2283 | 0.0617 |
| Rifampin | Azithromycin | 0.2790 | 0.0150* |
| Rifampin | Cefazolin | 0.0331 | 0.7636 |
| Rifampin | Chloramphenicol | 0.2942 | 0.0102* |
| Rifampin | Clarithromycin | 0.3571 | 0.0057* |
| Rifampin | Doxycycline | 0.3168 | 0.0251* |
| Rifampin | Erythromycin | 0.2983 | 0.0089* |
| Rifampin | Gentamicin | 0.0885 | 0.4331 |
| Rifampin | Imipenem | 0.1027 | 0.3705 |
| Rifampin | Oxacillin | 0.1017 | 0.4017 |
| Rifampin | Penicillin | 0.2135 | 0.0828 |
| Tetracycline | Amikacin | 0.4217 | 0.0012* |
| Tetracycline | Ampicillin | 0.4603 | 0.0002* |
| Tetracycline | Azithromycin | 0.0884 | 0.4442 |
| Tetracycline | Cefazolin | 0.0825 | 0.4607 |
| Tetracycline | Chloramphenicol | 0.0110 | 0.9242 |
| Tetracycline | Clarithromycin | 0.1432 | 0.2717 |
| Tetracycline | Doxycycline | 0.5888 | <.0001* |
| Tetracycline | Erythromycin | 0.0551 | 0.6334 |
| Tetracycline | Gentamicin | 0.5957 | <.0001* |
| Tetracycline | Imipenem | 0.1493 | 0.1990 |
| Tetracycline | Oxacillin | 0.0565 | 0.6460 |
| Tetracycline | Penicillin | 0.4239 | 0.0006* |
| Tetracycline | Rifampin | 0.1331 | 0.2522 |
| TMS | Amikacin | 0.2924 | 0.0226* |
| TMS | Ampicillin | 0.5430 | <.0001* |
| TMS | Azithromycin | 0.2608 | 0.0240* |
| TMS | Cefazolin | 0.2045 | 0.0651 |
| TMS | Chloramphenicol | 0.0863 | 0.4543 |
| TMS | Clarithromycin | 0.2149 | 0.0963 |

| Variable | by Variable | Kendall τ | Prob>\|τ\| |
|---|---|---|---|
| TMS | Doxycycline | 0.3108 | 0.0280* |
| TMS | Erythromycin | 0.2150 | 0.0610 |
| TMS | Gentamicin | 0.5272 | <.0001* |
| TMS | Imipenem | 0.1402 | 0.2245 |
| TMS | Oxacillin | 0.1703 | 0.1633 |
| TMS | Penicillin | 0.4965 | <.0001* |
| TMS | Rifampin | 0.0822 | 0.4764 |
| TMS | Tetracycline | 0.5378 | <.0001* |

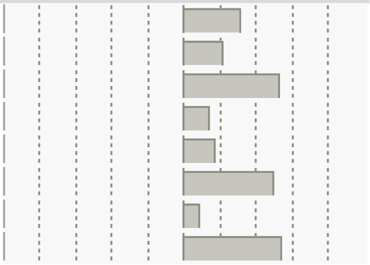

Supplement: Supplementary file 1 [file vetsci-10-00071-s001.zip › vetsci-2058149-supplementary.pdf]
